# Supplementary material for: Autoimmune Diseases and COVID-19 as Risk Factors for Poor Outcomes: Data on 13,940 Hospitalized Patients from the Spanish Nationwide SEMI-COVID-19 Registry
Source: J Clin Med. 2021 Apr 23;10(9):1844. doi: 10.3390/jcm10091844 (PMC8123043; doi:10.3390/jcm10091844)
Supplement: Supplementary file 1 [file jcm-10-01844-s001.zip › jcm-1151281-supplementary.pdf]

Supplementary Materials

Table S1. Baseline characteristics among groups.

	Without AD	Classical AD	Other AD	Miscelaneous AD	*p*-value
**Symptoms**					
Cough *n* (%)					
- No	3,547 (26.2)	54 (26.1)	40 (29.9)	6 (28.6)	0.909
- Dry cough	7,875 (58.2)	117 (56.5)	71 (53.0)	12 (57.1)	
- Chesty cough	2,103 (15.5)	36 (17.4)	23 (17.2)	3 (14.3)	
Arthromyalgia *n* (%)	4,041 (30.2)	52 (25.4)	35 (26.5)	6 (28.6)	0.386
Anosmia *n* (%)	874 (6.6)	14 (7.1)	8 (6.1)	3 (14.3)	0.553
Ageusia *n* (%)	7.5 (984)	12 (6.1)	12 (9.2)	2 (10.0)	0.730
Astenia *n* (%)	5,778 (43.3)	102 (50.2)	46 (34.8)	11 (52.4)	0.036
Anorexia *n* (%)	2,584 (19.4)	54 (26.9)	21 (16.0)	5 (23.8)	0.041
Sore throat *n* (%)	1,280 (9.6)	17 (8.4)	11 (8.3)	2 (9.5)	0.904
Headache *n* (%)	1,507 (11.3)	16 (7.9)	18 (13.6)	2 (9.5)	0.370
Fever *n* (%)	11,413 (84.4)	172 (83.5)	117 (87.3)	20 (95.2)	0.733
Dispnea *n* (%)	7,780 (57.6)	138 (67.0)	75 (56.0)	13 (61.9)	0.053
Diarrhea *n* (%)	3,122 (23.2)	60 (29.0)	31 (23.3)	5 (23.8)	0.284
Nausea *n* (%)	1,629 (12.3)	23 (11.4)	14 (10.6)	1 (4.8)	0.670
Vomiting *n* (%)	984 (7.3)	15 (7.3)	11 (8.3)	0 (0.0)	0.608
Abdominal pain *n* (%)	852 (6.4)	14 (6.8)	14 (10.6)	1 (4.8)	0.253
Confusion *n* (%)	1,596 (11.9)	24 (11.9)	17 (12.7)	0(0.0)	0.404
**Physical Examination Upon Admission**					
Systolic blood pressure (mmHg) (mean ± SD)	128.4 ± 21.3	129.1 ± 22.4	125.9 ± 21.9	129.1 ± 17.3	0.554
Diastolic blood pressure (mmHg) (mean ± SD)	73.7 ± 13.1	73.0 ± 13.6	71.1 ± 14.1	74.0 ± 10.6	0.119
Heart rate (bpm) (mean ± SD)	88.5 ± 17.5	87.6 ± 16.5	88.1 ± 17.5	87.7 ± 19.0	0.861
Temperature (ºC) (mean ± SD)	37.1 ± 1.0	37.1 ± 0.9	37.0 ± 1.0	36.9 ± 1.0	0.622
PaO2/Fi02(100) (mean ± SD)	287.1 ± 98.4	286.2 ± 100.4	286.3 ± 94.9	334.7 ± 99.8	0.350
Taquipnea >20bpm *n* (%)	4,078 (30.9)	66 (33.0)	50 (37.6)	4 (19.0)	0.209
Lung crackles *n* (%)	6,952 (52.7)	107 (53.2)	66 (50.8)	13 (65.0)	0.698
Lung wheezers *n* (%)	788 (6.0)	18 (9.0)	10 (7.8)	3 (15.0)	0.086
Lung roncus *n* (%)	1,424 (10.8)	18 (9.0)	22 (16.9)	2 (10.0)	0.124
**Lab Test Upon Admission**					
Hemoglobin (g/dL) (mean ± SD)	13.7 ± 1.9	13.1 ± 1.8	13.2 ± 1.9	13.9 ± 1.6	<0.001
Leucocytes (x10^6/L) (mean ± SD)	7,369 ± 5,575	7,360 ± 6,573	7,602 ± 4,540	6,343 ± 2,030	0.815
Lymphocytes (x10^6/L) (mean ± SD)	1,173 ± 2,204	914 ± 528	941 ± 508	1,048 ± 495	0.224
Neutrophils (x10^6/L) (mean ± SD)	5,508 ± 4,608	5,737 ± 6,202	5,900 ± 3,908	4,868 ± 1,676	0.609
CRP (mg/L)(mean ± SD)	86.4 ± 88.0	98.6 ± 107.9	92.6 ± 90.6	78.0 ± 84.5	0.198
Creatinin (mg/dL) (mean ± SD)	1.1 ± 0.8	1.2 ± 1.1	1.2 ± 1.0	1.0 ± 0.5	0.106
LDH (U/L) (mean ± SD)	367 ± 214	365 ± 187	379 ± 250	374 ± 176	0.939
Ferritin (mcg/L) (mean ± SD)	954 ± 1,106	645 ± 511	924 ± 1170	364 ± 243	0.022
D-dimer (ng/mL) (mean ± SD)	1,751 ± 7,334	3,088 ± 16,339	3,463 ± 13,484	798 ± 535	0.017
**Chest X-ray Upon Admission**					
Condensation *n* (%)	6,542 (48.8)	97 (46.9)	60 (44.8)	10 (47.6)	0.931
Interstitial infiltrate/ground glass *n* (%)	8,381 (62.5)	129 (62.3)	80 (59.7)	14 (66.7)	0.927
Pleural effusion *n* (%)	606 (4.5)	14 (6.8)	8 (6.0)	0 (0.0)	0.351

Data for patients with AD were imputed for sociodemographic variables, comorbidity and baseline treatment. Atherosclerotic vascular disease (ischemic heart disease, cerebral vascular disease, peripheral arterial disease). Non-ateriosclerotic vascular disease (heart failure, atrial fibrillation). Respiratory pathology (Asthma, COPD, chronic bronchitis, sleep apnea syndrome). AD: autoimmune diseases. COPD: chronic obstructive pulmonary disease. CRP: C-reactive protein; LDH: lactate dehydrogenase.

Table S2. Measures of effect, OR (95% CI),* of AD individually and immunomodulatory treatments prior prior to hospital admission with their propensity scores on the outcome variables$^{\alpha.}$

	Disease	Hydroxychloroquine	CS	cs/tsDMARDs	b-DMARDs
			**Mortality**		
SLE (23)	0.69 (0.11–4.15)	0.94 (0.13–6.73)	1.15 (0.31–4.26)	1.71 (0.45–6.50)	0.49 (0.06–4.14)
RA (113)	5.06 (1.06–24.17)*	0.56 (0.07–4.46)	0.81 (0.14–4.84)	1.62 (0.53–4.92)	0.22 (0.01–3.32)
PSS (19)	0.24 (0.04–1.33)	0.71 (0.37–1.38)	2.18 (1.59–3.00)*	1.12 (0.80–1.56)	1.88 (0.75–4.70)
SSc (13)	0.01 (0.00–0.11)*	0.64 (0.30–1.39)	3.60 (1.46–8.90)*	0.77 (0.44–1.34)	0.98 (0.64–1.51)
MTCD/Overlap (9)	0.83 (0.07–9.66)	0.37 (0.08–1.80)	2.53 (1.80–3.55)*	0.73 (0.34–1.57)	0.98 (0.62–1.55)
APS (4)	10.89 (0.91–130.98)	0.77 (0.41–1.47)	2.28 (1.83–2.83)*	0.97 (0.75–1.25)	1.05 (0.72–1.56)
IM (4)	0.74 (0.08–7.03)	0.78 (0.41–1.49)	2.28 (1.83–2.83)*	0.97 (0.75–1.25)	1.06 (0.72–1.56)
Vasculitis (25)	0.240 (0.03–1.65)	1.19 (0.52–2.73)	0.95 (0.36–2.50)	0.95 (0.36–2.50)	1.36 (0.81–2.29)
Spondyloarthropaties (33)	4.35 (0.84–22.49)	0.39 (0.05–3.09)	0.56 (0.09–3.30)	0.75 (0.29–1.94)	0.01 (0.00–0.18)*
PMR (48)	24.91 (6.29–98.56)*	2.61 (0.89–7.61)	0.96 (0.44–2.10)	1.17 (0.73–1.87)	0.54 (0.10–2.00)
PMR/Giant cell arteritis (57)	7.97 (2.51–25.32)*	2.23 (0.82–6.04)	1.51 (0.66–3.44)	0.09 (0.01–0.86)*	1.37 (0.75–2.49)
			**Composite Outcome**		
SLE (23)	0.51 (0.09–2.88)	1.40 (0.27–7.34)	1.13 (0.31–4.14)	1.58 (0.45–5.55)	0.44 (0.05–3.63)
RA (113)	3.84 (0.81–18.13)	0.90 (0.15–5.42)	0.85 (0.15–4.62)	1.49 (0.51–4.34)	0.22 (0.01–3.17)
PSS (19)	0.33 (0.08–1.36)	0.76 (0.39–1.47)	2.82 (1.34–5.94)*	0.80 (0.49–1.32)	1.82 (0.83–3.97)
SSc (13)	0.01 (0.00–0.08)*	0.83 (0.42–1.64)	2.90 (1.22–6.92)*	0.73(0.44–1.21)	1.15 (0.79–1.68)
MTCD/Overlap (9)	0.59(0.05–6.67)	0.50(0.11–2.16)	2.04 (1.49–2.80)*	0.72 (0.37–1.41)	1.16 (0.78–1.72)
APS (4)	7.77 (0.65–93.49)	0.97 (0.55–1.74)	1.88 (1.52–2.31)*	0.90 (0.71–1.14)	1.22 (0.86–1.74)
IM (4)	1.61 (0.24–10.69)	0.96 (0.53–1.71)	1.87 (1.52–2.31)*	0.90 (0.71–1.14)	1.22 (0.86–1.73)
Vasculitis (25)	0.19 (0.03–1.19)	1.32 (0.61–2.83)	0.85 (0.22–3.30)	0.90 (0.37–2.15)	1.83 (1.02–3.29)*
Spondyloarthropaties (33)	7.19 (1.74–29.64)*	0.44 (0.04–4.42)	0.34 (0.04–2.63)	0.61 (0.20–1.81)	0.01 (0.00–0.09)*
PMR (48)	18.30 (4.58–73.13)*	2.68 (0.96–7.47)	0.98 (0.50–1.89)	1.00 (0.64–1.54)	0.73 (0.20–2.60)
PMR/Giant cell arteritis (57)	7.79 (2.48–24.47)*	2.53 (0.90–7.12)	0.72 (0.37–1.43)	2.64 (1.0–7.00)	0.93 (0.48–179)
			**Complications During the Admission**		
SLE (23)	2.91 (0.51–16.64)	0.90 (0.18–4.55)	1.60 (0.61–4.17)	0.29 (0.12–0.72)*	8.63 (1.81–41.15)*
RA (113)	6.70 (2.10–21.36)*	0.54 (0.07–4.20)	1.17 (0.43–3.16)	0.20 (0.04–1.00)	4.19 (1.29–13.58)*
PSS (19)	0.18 (0.05–0.67)*	1.73 (0.48–6.23)	3.48 (1.51–8.02)*	1.13 (0.71–1.82)	1.38 (0.68–2.81)
SSc (13)	0.03 (0.00–0.21)*	1.02 (0.54–1.93)	3.21 (1.24–8.31)*	0.99 (0.62–1.59)	0.98 (0.68–1.42)
MTCD/Overlap (9)	0.30 (0.03–2.76)	0.61 (0.17–2.21)	2,.05 (1.49–2.83)*	0.89 (0.47–1,.68)	1.34 (0.83–2.16)
APS (4)	3.43 (0.29–41.29)	1.24 (0.72–2.13)	1.75 (1.43–2.14)	1.07 (0.87–1.33)	1.09 (0.79–1.52)
IM (4)	0.65 (0.11–4.09)	1.25 (0.72–2.18)	1.75 (1.43–2.15)*	1.07 (0.87–1.33)	1.10 (0.79–1.53)
Vasculitis (25)	0.18 (0.03–1.10)	0.71 (0.25–1.97)	21.02 (1.54–286.70)*	0.34 (0.12–0.96)*	1.12 (0.63–1.98)
Spondyloarthropaties (33)	3.42 (0.84–13.95)	3.69 (0.92–14.84)	0.44 (0.08–2.45)	1.72 (0.69–4.29)	0.01 (0.00–0.13)*
PMR (48)	1.69 (0.30–9.39)	5.13 (1.04–25.31)*	1.70 (0.85–3.39)	0.96 (0.67–1.38)	0.75 (0.37–1.54)
PMR/Giant cell arteritis (57)	1.78 (0.51–6.21)	2.74 (0.81–9.23)	1.34 (0.75–2.38)	3.41 (0.85–13.66)	0.75 (0.41–1.39)

* $p < 0.05$. α : Propensity scores was obtained with the inverse weighting method. Outcome variables were: mortality, composite outcome (mortality, mechanical ventilation, and ICU admission), and complications during the hospitalization. Predictor variables included in the logistic regression model to estimate the propensity score: age, sex, race, form of acquisition (common/nosocomial/residency), alcoholism, smoking, d**egree of dependence** (Barthel Index score), comorbidity, Charlson's index, immunosuppressive/immunomodulatory treatments prior to admission (anti-malarial, glucocorticoids, cs/ts- DMARD, b-DMARD) and during hospitalization (antimalarials, tocilizumab, immunoglobulins, anakinra, baricitinib, corticosteroids) and the presence of some complication during hospitalization (38 exposure variables to calculate the propensity scores). A total of 11,174 patients hospitalized by COVID-19 were included.AD: autoimmune diseases; APS primary antiphospholipid syndrome; b-DMARDs: disease modifying anti-rheumatic drugs, original biologic or similar; cs/ts-DMARD: disease modifying anti-rheumatic drugs, synthetic, conventional or targeted; ICU: Intensive Care Unit; IM: inflammatory myopathies; MTCD: mixed connective tissue disease; PMR: rheumatic polymyalgia; PSS: primary Sjögren syndrome; RA: rheumatoid arthritis; SLE: systemic lupus erythematosus; SSc: systemic sclerosis.
